# Editorial: Retina imaging in neurodegenerative disorders

**DOI:** 10.3389/fneur.2025.1613281

**Published:** 2025-05-06

**Authors:** Kai Jin, Jana Lizrova Preiningerova, Andrzej Grzybowski

**Affiliations:** ^1^Eye Center of Second Affiliated Hospital, School of Medicine, Zhejiang University, Hangzhou, Zhejiang, China; ^2^Zhejiang Provincial Key Laboratory of Ophthalmology, Zhejiang Provincial Clinical Research Center for Eye Diseases, Zhejiang Provincial Engineering Institute on Eye Diseases, Hangzhou, Zhejiang, China; ^3^Department of Neurology and Centre of Clinical Neuroscience, First Medical Faculty, Charles University and General Medical Hospital in Prague, Prague, Czechia; ^4^Institute for Research in Ophthalmology, Foundation for Ophthalmology Development, Poznan, Poland; ^5^Department of Ophthalmology, University of Warmia and Mazury, Olsztyn, Poland

**Keywords:** neurodegenerative diseases, retinal imaging, optical coherence tomography, early detection, neurodegeneration

## Introduction

The history of using an eye for diagnosing of central nervous system (CNS) disorders dates back to the 19th century. One of the pioneers of this Research Topic was Polish ophthalmologist Xavier Galezowski (1832–1907), who in 1866, published one of early textbooks on this subject and coined a term of cerebroscopy for this examination (1). The introduction of Optical Coherence Tomography (OCT) to at the end of 20th century changed Ophthalmology, but also enabled to analyze the nervous part of the retina with more details. In the late 90ties of the 20th century, Italian ophthalmologist Vincente Parosi, published the first studies on the RNFL in multiple sclerosis and Alzheimer's disease (2, 3). This opened a new chapter of the use of OCT in the evaluation of disease progression in numerous neurodegenerative disorders, including Multiple Sclerosis, Alzheimer's Disease, Parkinson's Disease, Huntington disease and related disorders, Friedreich's Ataxia, intracranial hypertension, Migraine, Neurosarcoidosis, Schizophrenia, Obstructive Sleep Apnea-Hypopnea Syndrome, Bipolar Disorder, and other Rare Neurological Syndromes (4).

Traditionally, these disorders are diagnosed based on clinical symptoms, but the retina, as an extension of the central nervous system, offers a unique, non-invasive window into neurodegeneration. Retinal imaging technologies, such as optical coherence tomography (OCT), adaptive optics, and fundus photography, provide high-resolution insights into the structural changes occurring in the retina that mirror the neurodegenerative processes in the brain (5). This editorial explores the role of retinal imaging in early diagnosis, disease monitoring, and understanding the pathophysiology of neurodegenerative diseases.

## Retinal imaging techniques and their role in neurodegeneration

Retinal imaging has emerged as a valuable tool in the diagnosis and monitoring of neurodegenerative diseases, offering a non-invasive means to visualize and quantify structural changes in the retina that reflect broader neurodegenerative processes in the brain. These changes in the retina can often precede clinical symptoms, making retinal imaging an essential modality for early diagnosis. Various advanced retinal imaging techniques, including optical coherence tomography (OCT), adaptive optics (AO), and fundus photography, provide high-resolution images of the retinal layers, enabling clinicians to assess structural and functional alterations that correspond with neurodegenerative changes in the central nervous system.

## Optical coherence tomography (OCT)

OCT is one of the most widely used imaging techniques in ophthalmology due to its ability to produce high-resolution cross-sectional images of the retina(6). In neurodegenerative diseases, OCT is primarily employed to measure the thickness of the retinal nerve fiber layer (RNFL) and the ganglion cell layer (GCL), both of which are directly affected by neurodegeneration. Studies have demonstrated that thinning of these layers occurs in several neurodegenerative conditions, including Alzheimer's disease and Parkinson's disease (7, 8).

In Alzheimer's disease, RNFL and GCL thinning, detected by OCT, has been correlated with amyloid plaque accumulation and brain atrophy, offering a potential early biomarker for disease progression (7). For Parkinson's disease, OCT imaging reveals changes in the retinal microvasculature and dopaminergic dysfunction, both of which mirror alterations in the brain's substantia nigra (8). Furthermore, OCT is being used to explore the retinal involvement in other neurodegenerative diseases such as Huntington's disease, where similar retinal changes are detected.

## Adaptive optics (AO)

Adaptive optics is another cutting-edge technology that allows for the direct imaging of individual retinal cells at an unprecedented level of detail (9). AO provides a higher resolution than OCT, making it particularly useful for observing the early stages of retinal degeneration and enabling researchers to visualize the cellular and sub-cellular structures within the retina. AO has been successfully employed to detect subtle changes in retinal microstructure that could precede observable loss in the retinal layers seen in OCT images. This includes the detection of ganglion cell loss and structural irregularities that reflect broader neurodegenerative changes in the central nervous system.

The ability to visualize individual photoreceptors, retinal ganglion cells, and capillaries with AO has led to the discovery of potential biomarkers for neurodegenerative diseases. For example, research has shown that early retinal changes in Alzheimer's patients, such as loss of retinal ganglion cells, can be detected using AO long before brain imaging reveals any significant changes. This has major implications for early diagnosis and intervention, providing an opportunity to slow disease progression through early therapeutic measures.

## Fundus photography

Fundus photography is one of the most common and widely available techniques used to capture the overall view of the retina. This technique is particularly valuable for identifying structural changes related to vascular alterations in the retina, such as retinal vessel narrowing, microaneurysms, and hemorrhages. These changes are not only indicative of diabetic retinopathy but also have implications for other neurodegenerative diseases, including Alzheimer's and Parkinson's diseases.

In Alzheimer's disease, for example, retinal vascular changes observed through fundus photography are thought to correlate with similar changes in cerebral microvasculature, providing an additional link between retinal pathology and neurodegeneration. Fundus photography has also been used to detect retinal changes in patients with vascular dementia, a condition that often coincides with cerebral small vessel disease. Furthermore, the ability to track vascular changes over time with fundus photography may help in monitoring disease progression, allowing clinicians to adjust treatment plans based on observed retinal changes.

## Other emerging techniques

In addition to the established imaging technologies, several other emerging techniques are being explored for their ability to assess retinal involvement in neurodegenerative diseases. These include fluorescein angiography, which is used to examine retinal blood flow, and optical coherence tomography angiography (OCTA), which allows for non-invasive visualization of the retinal microvascular network. These techniques have shown promise in identifying changes in the retinal vasculature that correlate with neurodegenerative diseases such as Alzheimer's and Parkinson's disease.

Furthermore, the use of multi-modal imaging approaches combining OCT, AO, and fundus photography is gaining traction, as it allows for a more comprehensive assessment of the retina, providing both structural and functional information. This holistic approach could improve the sensitivity of early detection of neurodegenerative diseases and offer a deeper understanding of the mechanisms driving these conditions.

## Role of retinal imaging in early detection and disease monitoring

The retina, being an extension of the central nervous system, offers a unique opportunity for early detection of neurodegenerative diseases. Since the retina is more accessible than the brain, retinal imaging can be used for regular, non-invasive monitoring of disease progression. The ability to detect retinal changes early, often before symptoms manifest in the brain, is a major advantage of retinal imaging in the context of neurodegeneration.

For instance, in Alzheimer's disease, retinal thinning, particularly of the RNFL and GCL, has been found to correlate with the extent of brain atrophy and cognitive decline. Similarly, in Parkinson's disease, changes in retinal nerve fibers and microvascular changes detected by OCT and AO have been linked to motor deficits. This provides a valuable opportunity for clinicians to intervene early, potentially slowing disease progression through early detection and personalized therapeutic strategies.

Moreover, the combination of structural retinal changes with other biomarkers, such as neuroinflammatory markers or genetic predispositions, can enhance the diagnostic accuracy of retinal imaging. Personalized medicine, driven by retinal imaging, holds the potential to revolutionize how neurodegenerative diseases are diagnosed, monitored, and treated.

## Challenges and future directions

Despite its potential, retinal imaging in neurodegenerative diseases faces several challenges. One of the key issues is the standardization of imaging protocols. The resolution of certain imaging techniques may not be sufficient to detect subtle, early-stage changes in the retina. Furthermore, while retinal changes can reflect neurodegeneration in the brain, the underlying pathophysiological mechanisms that link these changes remain unclear(10). Further research is needed to establish how retinal degeneration correlates with brain changes and to identify specific biomarkers for early diagnosis.

In addition, while retinal imaging provides valuable insights, its clinical application is limited by technical challenges and the need for expert interpretation of complex data. As imaging technology continues to improve, and as more research is conducted to elucidate the mechanisms linking the retina to neurodegenerative diseases, retinal imaging will likely become an essential diagnostic tool in clinical practice.

## Conclusions

Retinal imaging offers a promising avenue for the early detection, monitoring, and understanding of neurodegenerative diseases. By examining the retina, clinicians can identify biomarkers that reflect changes in the brain, allowing for earlier intervention and personalized treatment strategies. While challenges remain in the standardization of imaging techniques and the interpretation of data, the potential of retinal imaging to transform the diagnosis and management of neurodegenerative diseases is immense. Continued technological advancements and research are crucial to fully realizing the potential of retinal imaging as a non-invasive tool in neurodegenerative disease management.
